# Supplementary material for: Inheritable cell-states shape drug-persister correlations and population dynamics in cancer cells
Source: PLoS Comput Biol. 2025 Sep 19;21(9):e1013446. doi: 10.1371/journal.pcbi.1013446 (PMC12469175; doi:10.1371/journal.pcbi.1013446)
Supplement: Attachment — Submitted filename: Response to Reviewers.pdf [file pcbi.1013446.s002.pdf]

Reviewer Response

Additional Editor Comments :

The reviewers have undertaken a careful reading of the paper, raising important technical and conceptual questions. In particular, it is especially important to clearly establish the appropriateness of the data sets and analyses used in this work. The manuscript would also be improved by including a more thorough description of relevant statistics of the data and technical details of the methods.

Response: Thank you for organizing these Reviews, which have been very helpful. We are happy to see that the Reviewers are very positive about our work, and believe that it will be an important contribution to the field. We also appreciate the detailed comments by the Reviewers, which we now have addressed point by point. We have thoroughly revised the manuscript with details of the experiments, methods, new calculations, figures, Tables and updated results. We provide detailed responses below.

Reviewers' comments:
Reviewer's Responses to Questions

Reviewer #1:

In their manuscript "Inheritable cell-states shape drug-persister correlations and population dynamics in cancer cells", Iyer at all study the important problem of drug tolerant persister cells, which is germane to both cancer and infectious disease. Using rigorous modeling/inference and carefully done in vitro experiments, they support their conclusions well, and even challenge the model with data from single cell barcoding experiments. The writing is clear, the results support the conclusions, and the work provides an important springboard for future development/discovery. Honestly, I don't have any complaints. Well done.

Response: We thank the Reviewer for the very encouraging comments and are glad that he/she considers our results important for future discovery.

Reviewer #2:

In this very interesting and extensive manuscript, the authors utilize single-cell tracking from two cancer cell lines to investigate distribution of intermitotic times during cisplatin treatment. The authors link a theoretical framework for cellular growth with the single-cell data and population dynamics during treatment. Amongst a number of interesting conclusions, the authors conclude

that the time to division or death is not influenced by cisplatin treatment. The authors also use their theoretical framework to investigate recent results in colorectal cancer cell lines that used barcoding to study the distribution of drug-tolerant persistors in response to chemotherapy.

The manuscript reads as if it was written for a primarily biological audience and (possibly), a different journal (as there is no author summary, the bibliography is in a non-PLoS format and a few other formatting differences, including the bibliography). I find the manuscript suffers for the formatting decision to move all the technical and theoretical methods to the Supplemental Information and I struggled to understand the results using only the methods in the Main Text. Consequently, I suggest that the authors consider moving some of their theoretical work and model formulations to the main text.

Response: We thank the Reviewer for the precise summary, and the characterization of our work as "very interesting and extensive". We admit that we were concerned with including too much of the mathematical details in the main text, and kept a large part of it in the SI to appeal to a broader audience. However, at the Reviewer's suggestion, we have now moved some of the technical details into the Main Text methods section, while still trying to maintain overall readability. We explain these changes in more detail while responding to the subsequent comments by the Reviewer.

I have a number of questions regarding the current manuscript.

How were the probabilities of death or division events calculated and transformed into hazard rates for the age structured model?

Response: The hazard is defined as the ratio of the probability density and survival function (please see Section 8 of the SI for details). For the analysis before drug administration, the distribution of intermitotic times of the cells was fit to an Exponentially Modified Gaussian (EMG) by finding the set of 3 parameters of the EMG that gave the maximum data likelihood (details of likelihood construction is given in SI Section 3). This model fitting exercise provided the probability density function of the intermitotic time events, which in turn allows calculation of the survival function and hazard at any given instant in time. The same analysis was performed post drug on IMT and AT distributions to get the hazards for division and death respectively. The total hazard (for any event) post drug was then simply calculated as the sum of division and death hazards.

We have now added a brief explanation under Methods, section "Predicting population dynamics from single-cell measurements" of the Main Text.

How is the delay time for the drug estimated? As the authors are considering the intermitotic and apoptosis time distributions, I think it is important to explore how these distributions depend

upon the assumed drug-effect delay. How is this delay time implemented in the models and when estimating the IMT/AT?

Response: The way we model the effect of drug on the cells is via a hazard function for division post drug (which has different parameters than the hazard for division pre drug) and an entirely new hazard for death, which has no pre-drug equivalent. The delay time for the drug is modeled via a parameter T_{delay} (please see SI section 8 for details). The new, post-drug hazard functions are introduced only after a time T_{delay} post drug treatment, and we learn the value of this parameter along with all the other parameters describing the post-drug IMT and AT distributions using our MCMC procedure. Therefore, the IMT and AT distributions post drug are inferred from the data simultaneously with the delay time, and none of these parameters are being assigned based on prior assumptions.

The authors excluded a small population of cells that did not divide or die during the in vitro experiments. This decision, which is only stated briefly at the end of page 7, seems to be a fairly strong imposition on the cell-line data that the authors are modelling. In effect, one of the main results of the manuscript is that a single exponentially perturbed Gaussian distribution is sufficient to characterize the intermitotic times of the cell populations. As the authors have discarded data from a sub-population of cells that are slowly dividing (and thus likely have IMTs following a different distribution), the conclusion that a single IMT is sufficient to describe the data seems to follow directly from this assumption. I would be very interested to see how the results may change if these cells that do not divide or die are not discarded from the analysis (their IMT or AT could be incorporated as censored data.)

Response: We thank the Reviewer for raising this important point and we agree that we should have better explained our reason for discarding a small group of cells. We had originally only briefly mentioned the following: "*In both datasets there were a small fraction of cells which never divided from the beginning of tracking, and which did not show any bias towards end fate (survival or death post cisplatin). We*
excluded these cells from all further analysis." We explain this reason below in more detail with the actual numbers:

In the HCT116 dataset, we excluded a total of 18 lineages out of a total of 83 lineages in the dataset. Detailed reasons for exclusion of each lineage have now been added to the SI in Table1, Section 1.
Out of these 18 excluded lineages, we had a total of 13 cells that did not divide in their lifetime (never-dividers). 10 out of these ended up dying post cisplatin while 3 survived till the end of the experiment. The lower number of survivors is simply explained by the fact that the cisplatin concentration was slightly higher than its IC50 value and overall we saw about 66% cell death in the entire cell population. This implies that the never-dividers are unlikely to be enriched either for cells in the sensitive (*S'*) or pre-DTP (*P'*) state. Therefore, by excluding these cells, we are not artificially excluding more cells belonging to one cell state over the other, or introducing any bias in the cell cycle time distributions describing *S'* versus *P'* states. Therefore our main result,

that there is no measurable difference in the cell cycle time distributions of the ancestors of persisters versus sensitive cells will not be affected by discarding these 13 cells.

An identical argument holds for the U2OS cells, where 2/9 never-dividers died post low cisplatin, 8/13 died post medium cisplatin and 3/9 died post high cisplatin. Once again, these numbers show that the never-dividers are unlikely to be biased towards either cell death or persistence post cisplatin.

Additionally, since all of these non-dividers are both left and right censored, it is challenging to make any reasonable estimates of their cell cycle time, in the absence of any additional information of cell cycle phase markers. It is also possible that these are quiescent cells in the G0 phase, and including them in estimating the cell cycle time distribution may not be reasonable. Therefore, (1) given these cells will not change our result on cell cycle time differences between *S'* and *P'* states as explained above, (2) the fact that the numbers of non-dividers are very low, and (3) cell cycle time estimation is challenging if not impossible, we decided to discard this group of cells from any further analyses.

We have now added this explanation along with the numbers in the Main Text and SI. We have also added two Tables (current Tables 1 and 2) describing all the numbers in detail. Finally, we have also added Table 1 in the SI giving detailed explanations for why 18 lineages were excluded in the HCT116 dataset.

The HCT116 cell line is stated as starting with an initial concentration of 65 cells, but figure 1b shows a population of 1 cell; the initial population size is ~0 = log (1). Further, the authors show the single and mixture model for the IMT distribution in Figure 1e without the data. It's hard to evaluate the fit and I would appreciate seeing the (scaled) raw histograms.

Response: We are sorry for the confusion. As the Reviewer correctly pointed out, we had originally shown the average number of cells within a single lineage as a function of time, hence the y-axis started from near 0 in Figure 1b. In the HCT116 cells, we had a total of 65 lineages that were included in all analyses, but out of those only 49 were present at T=0 while 16 appeared a few time frames later. We have now changed Figure 1b, Figure 2a, and SI Figure 1 to show the starting point at log(49) = 3.9. We have also added this clarification in Table 1 and SI Section 1 by explicitly stating these numbers. We have also added the histogram to Figure 1f as requested by the Reviewer.

The authors state that using the inverse of the mean of the IMT distribution as a population growth rate does not lead to a good fit with the experimental growth curve and show this in Figure 2. I found this result difficult to parse. The net population growth rate, which is observed as the log(number of cells) involves the net proliferation rate. This net growth rate involves both cell proliferation and cellular loss due to apoptosis. In this respect, it isn't surprising to me that a

net growth rate corresponding to only the mean IMT (and not capturing apoptosis) would over-estimate the true growth curve. Can the authors better situate this result, or more clearly state how they estimated the proliferation rate from the IMT distribution? (Here, I found the manuscript difficult to follow, as the mathematical details are all in the S1 text.)

Response: All the results pertaining to Figure 2 correspond to the pre-drug scenario where there is very little cell death. More specifically, among the 65 lineages of HCT116 cells that we analyzed, there were no cells that died before cisplatin treatment. For U2OS only 6/247, 9/262 and 11/316 cells died before cisplatin treatment in the low, medium and high treatment arms respectively. Therefore around 3% or less cells died in these experiments when no drug was present. Therefore we used only the IMT distribution to predict population growth rate. The reason why the inverse of the mean does not do well is because it does not account for the variance in the IMT distributions. The Euler-Lotka equation on the other hand does account for the variance, hence does much better.

When we analyze the data post cisplatin treatment however, we do include both the IMT as well as the death time distributions to predict the population dynamics.

We have now added a clarification in the Main Text Section “Proliferation rate of cancer cells can be predicted from single-cell division times.”

The authors also repeatedly remark on the “error” between the model and the data. How is this error calculated and defined? Further, they state that using the inferred starting age distribution can “account for 4%” of the error. I don’t know how to understand this statement; how can the authors determine this?

Response: The error between true and predicted growth rates was calculated as follows: first the true population growth rate was calculated from the slope of the ln(number of cells) versus time plot obtained from the experiment. The estimated/predicted growth rates were computed from single-cell data using different methods – inverse of the mean or Euler-Lotka or the competing-risks effect model. The percentage difference between true and predicted rates are the “errors” reported in our manuscript.

The Euler-Lotka result assumes that the cellular population has reached a steady-state in terms of age distribution at any given point in time. However, this assumption may not be entirely valid in the experimental data, where the age-distribution of the cells at the time of seeding is likely to be different from this steady-state age distribution. This would then lead to some errors in the Euler-Lotka predicted growth rate versus actual growth rate. To assess how much that error might be, we simulated the whole cell division process on the computer, starting with an age-distribution that is not the steady-state age distribution. As explained in the manuscript, we inferred the starting age distributions from the HCT116 dataset (Fig. 2b) and the U2OS dataset

(Fig. 2e) and used these distributions as initial conditions in the simulations. Then we calculated the errors purely using this simulated data, and found it to be about 4% for the U2OS dataset.

We hope this clarifies the Reviewer's doubt. We have now added a clarification on the definition of the "error" in the Main Text Section "Proliferation rate of cancer cells can be predicted from single-cell division times."

On line 274, the authors note that the decay rates are "distinctly" different between high, low, and control cisplatin treatments. Are these differences statistically significant? Leading to their conclusion that the times to division or death post treatment are not treatment dependent as the IMT and AT distributions only showed "negligible differences". What does negligible difference mean in this context? Can the authors quantify this statement (both in terms of the decay rates and the "negligible differences" between distributions). Without these differences being cleared defined and reported, it is very difficult to evaluate the claim that these results contradict the long-standing assumption that cytotoxic drugs effect proliferation or apoptosis rates.

Response: The linear regression fits to the population decay provide both the slopes and the error in the slopes. By "distinct", we meant that after accounting for the errors, the slope +- error values do not overlap between low, medium, and high cisplatin concentrations. We show the values here in the table below and report them (with correct significant digits) in the Main Text. Further, note that the decay rate at high cisplatin is about 3 times the rate at low cisplatin.

Value	High	Medium	Low
Slope (growth rates)	-0.0249571	-0.0119543	-0.008813
Std Err	0.0003761	0.0001905	0.000132

For the distributions of IMT and AT, we performed the Kruskal Wallis test for both IMT and AT distributions:

Results IMT
- chisq statistic (df=2) = 2.99
- p value = 0.2237

Results AT
- chi-squared = 1.2658, df = 2,
- p-value = 0.531

These results show that the medians of the IMT distributions are not significantly different across drug concentrations. This is true for the AT distributions as well.

We have now added these details in the Main Text under Section “First exponential decay of drug treated cancer cells is not established by single-cell division and death times”, and in the caption of Figure 3c,d.

On lines 301, the authors remark on a significant “right skew” in the inferred IMT distribution compared to the measured IMT (slower division times.) Is this necessarily the case? I can imagine two distributions with the same mean division time but different 3rd moments. I found it difficult to evaluate this result without a precise mathematical statement.

Response: We apologize for using the word “skew” loosely. We were simply referring to the longer right tails of the inferred IMT distributions, which are obvious from the larger spread of the x-axis values. We have now corrected this in the Main Text.

I was unable to follow the final result section, on barcode labelling in simulations, using the methods in the manuscript. Here, I think the manuscript would benefit from moving much of the technical details currently in the SI to the main text, as it would allow the authors to make precise statements regarding their models that are currently missing.

Response: For the barcoding simulations, we used Model *M3* with the final parameters obtained by matching the lineage correlations and decay rates measured in the HCT116 experiments. To mimic barcodes, we simply provide lineage numbers to the initial ancestor cells that the simulations are started with. These lineage numbers are then maintained across cell divisions, to mimic the inheritance of barcodes.

We have added a clarification on how the barcoding simulation works in the Methods section of the Main Text under the description of Model *M3*.

I took a quick look at the listed Github page and could not find a relevant repository for this manuscript. I appreciate that the authors state that the code will be available on Github, will they also make the underlying data publicly available?

We have provided both the data as well as the code on the Github link:

Reviewer #3:

Iyer et al.: ““Inheritable cell-states shape drug-persister correlations and population dynamics in cancer cells””

Summary:

The authors combine time-lapse microscopy data from two cancer cell lines with quantitative modeling to explore the relationship between single-cell kinetics and population dynamics in drug-tolerant persisters. Their key finding is that while single-cell division and death time statistics accurately describe population dynamics before drug exposure, they fail to do so afterward. Surprisingly, they observe that increasing drug concentration leads to a threefold increase in population decay rates, despite no significant changes in single-cell division or death times. To reconcile these observations, the authors propose and validate a model in which cell fate decisions are largely predetermined by pre-existing cellular states inherited across 2–3 generations, rather than being dictated solely by birth and death rates post-drug exposure. They further support this model with orthogonal barcoding data and fluctuation test analysis.

Response: We thank the Reviewer for this succinct summary of our work.

Overall assessment

The manuscript is well-written, engaging, and addresses a highly relevant topic. The authors' data-driven approach is appropriate, insightful, and presents a novel perspective on cancer persister dynamics. Their quantitative analyses appear methodologically sound.

A key strength of this study is its integration of both single-cell and population-level data to inform quantitative models—a relatively novel approach that provides valuable insights into the principles underlying persister cell dynamics.

That said, there are some aspects of the study that would benefit from further clarification and refinement. Indeed, while the authors’ findings have the potential to be highly impactful, I have significant concerns regarding the extent to which the authors' claims are supported by the quality of the data and the pertinence of their analyses and models.

Response: We are glad that the Reviewer thinks our work provides a novel perspective on persister dynamics by integrating single-cell and population dynamics of cancer cells. We also thank the Reviewer for the very careful reading of the manuscript and raising all the important points of concern, which we have now addressed point by point.

One key point concerns the datasets used. The U2OS dataset, as acknowledged by the authors, does not exhibit biphasic death kinetics—a hallmark of persister cell dynamics. Additionally, partial lineage tracking in this dataset raises further concerns about its appropriateness. Given that this dataset, specifically its concentration-dependent behavior, appears to play a crucial role in the inference and validation analyses, it would be helpful for the

authors to explicitly explain why the U2OS dataset remains relevant for understanding persister dynamics despite its apparent a priori limitations.

Response: We thank the Reviewer for this question and for correctly pointing out that the U2OS dataset does not show the second exponential decay phase (presumably because U2OS is a slower dividing cell type, and 3 days post drug was not sufficient to detect the second phase). It is also correct that only one randomly chosen sister cell was tracked in this dataset, unlike in the HCT116 data where all cells were tracked. However, even though these may appear as limitations, the U2OS dataset remains relevant and crucial for this study for the following reasons:

(1) The main mathematical finding of this study is that while the IMT distribution is sufficient to predict the pre-drug growth rate (within 10-20% error), the post-drug IMT and AT distributions cannot predict decay rates, with consistent errors of ~100%. This important result is established using the *first* exponential decay phase only. Hence the absence of the second exponential decay phase in U2OS does not affect this analysis.

(2) The lack of full lineages does not matter for inferring the IMT and AT distributions. Indeed, the correct statistical way of inferring these distributions is to choose just one randomly selected daughter of any given mother cell, as has been discussed in many previous studies. Therefore the important result of this study comparing the post-drug IMT and AT distributions for different cisplatin concentrations is not affected by the lack of full lineages. Indeed, this comparison could only be made in the U2OS cells as the HCT116 data was generated with only 1 drug concentration, making the U2OS data crucial for this work.

(3) Finally, even though the U2OS cells lacked full lineages, we could still utilize mixture models to look for evidence of multiple cycling populations in the pre-drug scenario.

In summary, in spite of the apparent limitations of the U2OS dataset, it was still crucial for our study for the above three reasons. We have now added a brief explanation of this point in the Main Text under the section “Subpopulations with distinct proliferation rates cannot be detected in HCT116 or U2OS cells.”

Furthermore, the lack of clear discussions regarding the data statistics (e.g. number of cells considered in the analyses), and the specific details of the tested models, makes it difficult to fully assess the robustness of the model selection analysis. Comparisons between experimental data, predictions from different models, and simulations with similar statistical constraints are not consistently provided. Additionally, other potential explanations for the authors' findings are not always considered. I believe that a more thorough examination of these aspects would strengthen the authors' claims and provide better support for their conclusions.

Response: We have now addressed these points in detail. We have provided two new Tables in the main text (Tables 1 and 2 in the current manuscript) which provide detailed statistics on the cell numbers used, discarded, etc. Additionally, we have provided a new Table in the SI (Table 1) detailing the reasons for discarding certain lineages in the HCT116 dataset. These tables are now accompanied with better descriptions of the datasets both in the Main Text as well as in the SI.

Furthermore, we have now provided more details on the statistical tests used to compare IMT and AT distributions, and also details on error bars on the slopes of the population dynamics. A summary of these changes are provided below, as well as in Response to the previous Reviewer's questions:

The linear regression fits to the population decay of U2OS cells provide both the slopes and the error in the slopes. To show they are distinctly different, as we had claimed in the original manuscript, we now show that the slope +- error values do not overlap between low, medium, and high cisplatin concentrations. We show the values here in the table below and report them (with correct significant digits) in the Main Text.

Value	High	Medium	Low
Slope (growth rates)	-0.0249571	-0.0119543	-0.008813
Std Err	0.0003761	0.0001905	0.000132

For the distributions of IMT and AT, which we had originally claimed to be essentially indistinguishable, we now show the results of the Kruskal Wallis test for both IMT and AT distributions:

Results IMT
- chisq statistic (df=2) = 2.99
- p value = 0.2237

Results AT
- chi-squared = 1.2658, df = 2,
- p-value = 0.531

These results show that the medians of the IMT distributions are indeed not significantly different across drug concentrations. This is true for the AT distributions as well.

We have now added these details in the Main Text under Section "First exponential decay of drug treated cancer cells is not established by single-cell division and death times", and in the caption of Figure 3c,d.

Another key point regards the rationale for testing certain models, which is not always evident to me. A key contribution of this study, as I said, is its combined analysis of both single-cell and population data. As such, it is not a priori clear why model M1, for example, should be considered as a viable alternative, given that it assumes persister cells continue growing during treatment and does not seem to reproduce biphasic death kinetics. If some models are a priori clearly incompatible with data, then the model selection analysis—though methodologically sound—becomes somewhat less meaningful. More informative comparisons would involve models that are thought to plausibly capture all key experimental findings.

Response: We thank the Reviewer for this very insightful question and for pointing out the limitation of Model M1 in the current manuscript. It is indeed true that Model M1 in the form originally presented, will not generate a distinct second exponential phase of the characteristic biphasic kinetics, as the Reviewer correctly points out. This would be the case because cells in the persister state (*P'*) only proliferate post drug treatment, resulting in an almost immediate rebound in growth after all sensitive cells die. (We had however still considered Model M1 as a valid alternative model because it recapitulates the experimentally observed lineage correlations in fate).

To address this issue, we have now made a modification to Model M1 while retaining the core idea behind the model, which is to have all post-drug cell fates decided before drug addition, based purely on the cell state. The modification we introduce is that cells in the *P'* state are now allowed two fates post drug, divide or stay quiescent without dividing. With this change, we can now get a distinct second phase of the classic biphasic kinetics as we show in the example plot below.

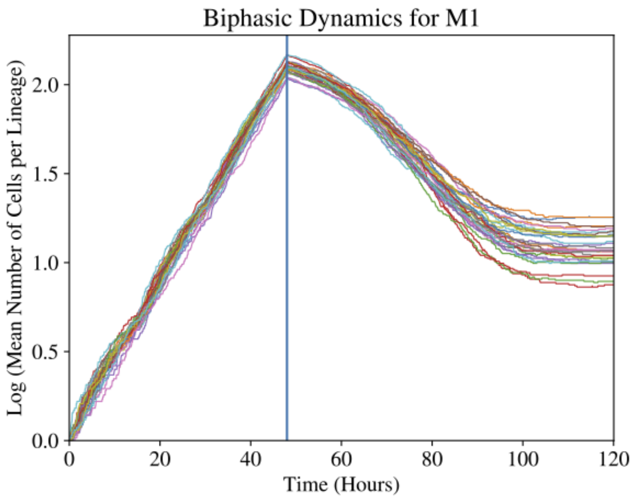

However, even though this updated Model M1 can now capture both the lineage correlations in fate and the biphasic kinetics post drug treatment, it still cannot explain the origin of different population decay rates (of the first exponential decay phase) for different drug concentrations.

This is simply because every cell is already fated to either die, or survive/divide based on its cell state at time of drug addition. The drug itself plays no role in determining fate, and hence neither does its concentration play any role.

Therefore, our original conclusion still remains unaltered, and the argument is that one needs to have contributions to cell fate decisions *both* pre and post drug, as described by the final model Model M3.

We have now updated the details of the new Model M1 in the Main Text Methods, and have also added a few lines in the Results section to explain why M1 is a viable alternative. We have also added the biphasic plots generated by Model *M1* in the SI Section 9.

In this reviewer's opinion, these concerns represent a significant limitation of the study in its current form. This is particularly important given that some of the study's conclusions challenge established knowledge in the field. In such cases, it is crucial that claims be supported by robust data and rigorous quantitative analyses. While the manuscript acknowledges some potential limitations, a more explicit discussion of these aspects would improve clarity and enhance the study's overall impact.

To strengthen the manuscript and ensure its findings are as compelling as possible, I would encourage the authors to undertake substantial revisions addressing these points.

I therefore recommend a major revision of the manuscript.

Response: We have now extensively revised the manuscript by adding detailed tables with the statistics of the datasets in both Main Text and SI, clearer explanations of the Models via a separate Figure, new calculations of Model M1 that allow it to create biphasic kinetics, a complete reanalysis of the Luria Delbruck test for both experiments and model, and updated explanations throughout the manuscript.

%%

Specific comments.

1. The manuscript should more effectively and transparently communicate the nature and quality of the data and models considered. While clearly written, it lacks a structured and easily accessible presentation—both textually and visually—of the datasets analyzed. Despite these data originate from the authors' previous work, I strongly recommend adding a dedicated Methods section, along with a corresponding main figure, to explicitly detail the dataset's key aspects, including the number of cells analyzed, experimental protocols (briefly), and statistical properties.

Response: We have now added 3 new Tables with details of the datasets involved – 2 in the main text and 1 in the SI. We figured that all these details would be hard to show in a figure, hence we resorted to Tables. We have also explained these datasets more thoroughly, both in the Main Text as well as the SI.

Since the study's conclusions heavily rely on these datasets, it is crucial that readers can readily access and interpret this information. Currently, relevant details are dispersed across the Results and Supplementary Information sections, making them less accessible. Beyond the infographics in Figure 1A–D, no other visual representation of the data is provided. A dedicated figure summarizing the dataset could, for instance, incorporate Figure 1A, B, D, and E, supplemented with additional panels outlining the experimental protocol and statistical power. This addition would significantly improve clarity and ensure that the role of the data in shaping the study's conclusions is immediately evident.

Response: As we mentioned in response to the previous comment, we have now added all the details of the datasets in Tables, along with better explanations in the Main text and SI.

Similarly, Figure 4A, which presents the different models considered, should be extracted and presented as a separate figure dedicated to model descriptions. This would enhance readability and help distinguish between the data, model and results components of the study.

Response: We thank the Reviewer for this helpful suggestion and have now implemented this change. The new Figure 4 now comprises only the 4 Models, *M0 – M3,* with updated images to reflect additions made during the Review process as well as more careful depictions of the models. The caption of the new Figure 4 also more clearly lays out the differences between the models.

---

2. Can the authors compare model M3 with a more competitive alternative than models M1 and M2? Alternatively, could the authors clarify why M1 and M2 are considered competitive alternatives? For instance, while the authors present their findings as contrasting a more predominant drug-induced scenario, it seems to me that neither model M0 nor models M1 and M2 effectively capture a drug-induced scenario where susceptible cells transition to a persistent state exclusively during treatment and in a drug-dependent manner.

Response: The reason the original Models *M1* and *M2* were introduced in the manuscript was to demonstrate that each one captures a certain aspect of the experimental observations, but not all. *M1* captures lineage correlations but not drug concentration dependence of decay rates, while *M2* can explain drug concentration effects but not lineage correlations in fate. Therefore we finally introduced Model *M3* by essentially combining *M1* and *M2*, which allowed explaining both the lineage correlations in fate as well as the drug concentration dependence of the decay

rates. *M1* and *M2* were not envisioned as “competitors” of *M3*, but rather as stepping stones towards building up a minimal model with all the essential components.

Having said that, we have now updated our model *M1* based on the Reviewer’s valid criticism that it cannot generate distinct biphasic kinetics. The updates and results have been presented in more detail above, in response to the Reviewer’s last Major Comment.

If we understand the Reviewer correctly, Model *M2* in essence is indeed capturing the scenario mentioned by the Reviewer: “susceptible cells transition to a persistent state exclusively during treatment and in a drug-dependent manner”. In Model *M2*, there is no notion of different cell states before drug administration, and all cells are equal. At time of drug, fates are assigned randomly to the cells, with more probability of death compared to survival for higher drug concentrations. This is effectively modeling a scenario where persister cells emerge exclusively after drug treatment, in a concentration dependent manner. While the model doesn’t have explicit “states”, this does not really matter, and conceptually one could think of all cells before drug administration as “susceptible cells” and that after drug, some are transitioning into persisters.

We have now added a short explanation of this point in in Main Text, under Results Section “Cell-state inheritance along with fate decisions before *and* during drug treatment control cancer population dynamics.”

---

3. “Subpopulations with distinct proliferation rates cannot be detected in HCT116 or U2OS cells” --> I would specify that this result applies **before treatment**. Same for the next result section “Proliferation rate of cancer cells can be predicted from single-cell division times”

Response: We have updated both the Results section headings now.

---

4. On line 191 the authors state “we identified persisters as those cells surviving till the of the experiment”. While this is reasonable, can the authors validate this claim by quantifying, for example, the fraction of persister cells predicted by a given model at that point?

Response: As suggested by the Reviewer, we have now quantified the fraction of persister cells per lineage at the last time points of both the HCT116 dataset and the Model *M3* simulation. The distributions of these fractions are shown in the Figure below, and there is a reasonable match between the experimental data and Model *M3*. We have now added this Figure to our new updated Figure 6.

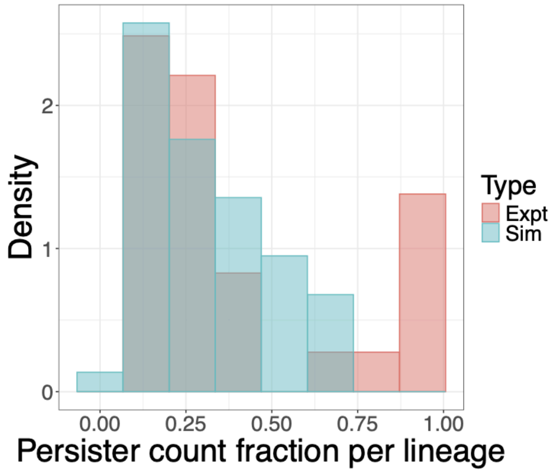

---

5. In Figure 1C, the authors conclude that if persisters pre-existed treatment, they should have no fitness cost relative to susceptible cells. While I agree with this reasoning, how can the authors rule out the possibility that, in their data, no persister cells actually pre-exist treatment (i.e., that Figure 1C represents only one cell type=susceptible)? If transitions between the two states can occur between consecutive birth and death events, could it be that a persister cell's ancestor was actually a susceptible cell? Would it be possible to validate this "ancestor-type reconstruction" analysis through simulations?

Response: We thank the Reviewer for this very insightful question. We respond first with a qualitative, and then with a more quantitative argument.

Qualitatively, if there were no cells in the *P'* state (pre-DTP) before drug administration and all persisters
arose from state-switching post drug (effectively Model *M2*), the lineage correlations amongst persisters would be hard to explain. One would have to argue that the drug somehow induces a coordinated cell-state switch from *S'* to *P'* state only for closely lineage related cells, which is not easy to justify. A simpler explanation is that *P'* cells already existed before drug, passed down the state to its progeny, which then survived post drug treatment leading to correlated cell survival on the lineages.

However, the Reviewer is entirely correct in suggesting that a small fraction of persister cells could indeed have arisen from a cell in the *S'* state that eventually survived (potentially because the drug induced a switch to the *P'* state). This is exactly the situation we modeled using Model *M3*, where we introduced the “Fate Matrix *F*”. *F* allows for some small probability of mixing in the state-to-fate map, i.e, a cell in *S'* state at time of drug goes on to survive, while a cell in *P'* state

at time of drug goes on to die. We ran quantitative simulations of this model, and though we did not do any formal inference, we did some coarse parameter sweeps to find the approximate parameter regimes where we could near-quantitatively explain all of our data. As we reported in Figures 4f and 4g (now Figures 5e and 5f in the updated manuscript), the probability of *S'* to survive was 0.03 while the probability of *P'* to die was 0.35. While not included in this manuscript, we are currently working on more accurate approaches based on constructing likelihoods for lineage trees to precisely infer these parameters from the data.

We have now included a discussion of this point under the Result section "Cell-state inheritance along with fate decisions before *and* during drug treatment control cancer population dynamics."

---

6. In Figure 2 and Table 1, the authors report that the population growth rate before drug addition can be predicted from the single-cell IMT distribution, albeit with higher estimation errors than expected from simulations of the age-structured model with similar simulated cell numbers. The authors suggest that this discrepancy may arise from artifacts in the image analysis pipeline or unaccounted biological factors, such as size-control effects during cell-cycle progression. Can the authors elaborate more on how size-control might influence their analysis?

Response: Control of cell cycle speed by hidden variables (which could include cell size; see Kuchen et al, eLife 2020) can generate a variety of interesting correlation structures in the cell cycle times of lineage related cells. Some of these, for instance mother-daughter correlations, in turn can contribute to the predicted population growth rate (Powell, 1956). The basic Euler-Lotka equation we use to predict population dynamics ignores any correlations amongst lineage related cells.

We have now added a line on this along with the eLife reference.

Additionally, could the observed discrepancy be partially explained by the omission of cell death in the analysis? It is unclear why cell death was neglected, given that the population growth rate being estimated is the effective growth rate—that is, the net difference between growth and death rates. Or am I wrong? Can the authors clarify the rationale for this choice?

Response: We think cell death is unlikely to explain any significant amount of the discrepancy that we report in Figure 2 and Table 1. This is because all the results pertaining to Figure 2 correspond to the pre-drug scenario where there is very little cell death. Among the 65 lineages of HCT116 cells that we analyzed, there were no cells that died before cisplatin treatment. This of course completely rules out cell death playing any role in the discrepancy in HCT116 cells. Even for U2OS, only 6/247, 9/262 and 11/316 cells died before cisplatin treatment in the low, medium and high treatment arms respectively. Since these numbers amount to only ~ 3% cell death in the absence of drug, we used only the IMT distribution to predict population growth

rate. Note however that when we analyze the data post cisplatin treatment, we do include both the IMT as well as the death time distributions to predict the population dynamics.

We have now added a clarification in the Main Text Section "Proliferation rate of cancer cells can be predicted from single-cell division times."

---

7. The finding that single-cell IMT and AT distributions remain unchanged across different drug concentrations is both surprising and intriguing. Can the authors clarify their rationale for including in this analysis the division times of cells born before drug exposure?

Response: The primary reason for having to include cells born before drug administration is because there are very few cells that are born after cisplatin exposure *and* go on to finish a full cell cycle to divide again. As we now report in Tables 1 and 2, there were no HCT116 cells that finished a full division event purely post cisplatin, while in the U2OS datasets there were just 2/232, 2/240 and 1/296 such cells in the Low, Medium and High concentrations respectively. This behaviour is typical in the case of cisplatin.

As we had shown in our original study (Chakrabarti et al, Nat Comm 2018), these "straddling cells" show a clear elongation of their full cell cycle time, demonstrating the action of the drug and the possibility of extracting the post-drug kinetics. To carefully disentangle the pre-drug versus post-drug IMT distributions, we carried out the inference procedure which is described in the SI Sections 7 and 8 of our current manuscript, with the results in Figures 3e-h.

We have now added this short clarification in the Main text Results section "First exponential decay of drug treated cancer cells is not established by single-cell division and death times"

If the drug primarily affects a specific phase of the cell cycle (e.g., G1 or S/G2/M), some of these cells may not experience the drug's effects and could divide as usual, potentially introducing a bias in the analysis.

Response: In our earlier study from where the HCT116 data was taken, we had ruled out potential connections between cell cycle stage at the time of cisplatin treatment and cell death (Supplementary section 3, Supplementary Figure 3 of Chakrabarti et al, Nat Comm 2018). We found that cells were equally likely to die irrespective of cell cycle stage at time of cisplatin administration. Therefore it is unlikely that the cells are not experiencing the drug based on their cell cycle stage. However, it is true that cells in G1 during cisplatin treatment were more likely to slow down and get arrested following treatment, than cells in G2/M (Supplementary Figure 3i of Chakrabarti et al, Nat Comm 2018). But we do not think these differences would affect the comparison of IMT and AT distributions across experiments varying the drug concentration. This is because at the time of drug administration, there would be roughly equal numbers of cells in each cell cycle stage across the different experiments. These are unsynchronized cells, which

means the fraction of cells in each stage will be roughly equal to the fractional duration of each stage, hence across experiments will be nearly the same at time of drug.

Would it be possible to replicate the analysis considering only cells born after drug exposure to assess whether this influences the results?

Response: We have explained in the point above why this cannot be done due to extremely low numbers.

Additionally, can the authors rule out the possibility that the drug concentrations used in this analysis are beyond the range where they still affect single-cell division and death rates? As the authors note, it is generally believed that the death rate increases with drug concentration, but at some point, this increase should plateau as the death rate reaches a maximal inducible value. Could the authors test this possibility by comparing model predictions with the observed concentration-dependent behavior at the population level? Notably, a concentration-dependent response at the population level could still emerge from concentration-dependent transition rates (e.g. from susceptible to persister cells).

Response: The drug concentrations used, for example in the U2OS study, ranged from below IC50 to above IC50 of cisplatin. This falls in the region of an `S' shape dose-response curve where the cells are
most responsive to the drug. Similarly, in the HCT116 dataset cisplatin was administered at IC50. Hence it is very unlikely that we are in any sort of saturating regime.

We agree with the Reviewer's last point regarding a concentration-dependent response at the population level emerging from concentration-dependent transition rates, which is precisely what the fate matrix `*F*' models in our Model *M3*, which we argue is the best description of reality among models *M0-M3*. As we have previously explained in response to one of the Reviewer's earlier questions, the fate matrix allows some small probability of every susceptible cell at the time of drug addition to attain a persister fate post drug, in a concentration dependent fashion. For instance, from the coarse parameter sweeps to match the HCT116 data, we found that this susceptible state → persister fate probability is about 0.037.

---

8. Regarding the fluctuation test analysis, could the authors clarify in the main text how the number of persisters was calculated experimentally? Specifically, does it refer to the number of surviving cells in each lineage at the end of the experiment?

Response: The Reviewer is correct – the number of persisters per lineage is simply the number of cells surviving within each lineage at the last time point of the experiment.

Additionally, it seems to me that the pre-existing and drug-induced scenarios do not necessarily imply super-Poissonian and Poissonian statistics for the number of persisters, respectively. The specific statistics followed by the number of persister cells depends on the assumptions made about their dynamics both before and after drug exposure. For example, if persisters neither grow nor die, their number will follow a Poisson distribution, even if they were generated before drug exposure (as their evolution would be a counting process, similar to mutational events in the standard Luria-Delbrück setup). On the other hand, if persisters can grow, their number at a given time after treatment would follow super-Poisson statistics, even if they are generated only in the presence of the drug.

Therefore, it seems important to me to note that the super-poissonian statistics of persisters predicted by model M3 (cf. Fig. 4A) arise specifically because this model assumes persisters can divide during treatment. However, as discussed, this does not necessarily rule out a model where persisters are drug-induced (i.e., generated only after drug exposure) and also can divide during treatment. Can the authors test for this scenario?

Response: This is a very interesting and subtle point and we thank the Reviewer for raising it. We agree that any cell division post drug needs to be treated carefully, and that even if all persisters arise purely post-drug but proliferate in the presence of the drug, then this scenario would also generate a super-Poissonian signature. Therefore just finding variance/mean > 1 is not sufficient to argue about the timing of persister development, as the Reviewer correctly points out. We have therefore carefully reanalyzed both the experimental data as well as the simulated Model *M3*, to account for post-drug cell divisions in the calculation of the variance/mean factor.

To get rid of the effect of divisions post drug, we now carry out the following procedure for analyzing both the experimental and simulation datasets: we first identify all the extant cells at time of drug administration. We then track their post-drug fate. If any of these cells divide post-drug, we randomly select only one daughter from this division event. Therefore, we record the final fate of as many cells as the number that was extant at time of drug administration. The rest of the analysis is as before, where we identify the number of persisters per lineage, and then calculate the variance/mean (and error bars using bootstrap).

The comparison of the measured variance/mean needs to be performed not against a Poisson distribution (variance/mean =1), but against a binomial distribution (variance/mean = 1- p) due to the small number of cells involved. For our HCT116 experiments, p, the probability of getting persisters at the end of experiment, is 0.36. Therefore the variance/mean expected from a purely drug-induced scenario is not 1, but 0.64. From the data and the new analysis, we get variance/mean = 2.4 while from Model *M3* we get 1.6. Both these numbers are higher than 0.64, and thus the conclusion still remains that there is a clear decision making process before cisplatin administration that is giving rise to proliferating persister states.

We have now rewritten the Luria Delbruck section completely, to include these modified results and analyses procedures.

Given these considerations, I recommend that the authors provide more precision in describing the models considered in their comparisons and model selection analyses.
