# Supplementary material for: Inheritable cell-states shape drug-persister correlations and population dynamics in cancer cells
Source: PLoS Comput Biol. 2025 Sep 19;21(9):e1013446. doi: 10.1371/journal.pcbi.1013446 (PMC12469175; doi:10.1371/journal.pcbi.1013446)
Supplement: Attachment — Submitted filename: Response to Reviewers - Round 2.pdf [file pcbi.1013446.s003.pdf]

Response to Reviewers – Round 2

Reviewer #2: I appreciate the authors effort in their response and revision, which has addressed many of my earlier concerns. I have a few remaining questions regarding the results and methods in the revised manuscript. As in my first review, I found the methods in the Main Text inadequate to understand the results. As PLOS Comp Biol does not have a page limit and I believe the manuscript will be significantly easier to understand if the mathematical and technical details are presented in the main text, I suggest that the authors consider moving some of their theoretical work and model formulations to the main text.

Response: We thank the Reviewer for the positive comments on the revised manuscript, and also for this renewed suggestion of including more modelling details in the Main Text. We have now moved much of the mathematical details, specifically for Models *M0* and *M3*, an analytical calculation explaining the competing risks effect, and the barcoding analysis to the Main Texts Methods section.

In the response to my earlier comments, the authors state
"among the 65 lineages of HCT116 cells that we analyzed, there were no cells that died before cisplatin treatment. (with a similar comment regarding the other cell line)"
While I appreciate that this finding strengthens the use of the inverse of the mean of the IMT distribution for comparison against the Euler-Lotka equation, it seems contradictory with the authors conclusion that
"This result contradicts the widely used assumption that the effect of drugs can be well described either as increasing the cellular death rate or reducing the division rate [lines 341 & 342]."
It could be that I'm missing something simple, but I would appreciate clarification on how these two statements can be reconciled (especially as there are certainly cells that die post treatment, as shown in Table 1 & 2).

Response: The two statements pertain to different scenarios – the first statement on the absence of cell death is for the pre-cisplatin scenario, while the second statement on the failure of Euler-Lotka in predicting population dynamics is for the post-cisplatin scenario. In the absence of drug, Euler Lota is capable of predicting population dynamics from single cell measurements. It fails only in the presence of drug, when significant cell death occurs.

On lines 264-265, the authors conclude that "best explained by a single population of cycling cells,
265 not by multiple sub-populations with distinctly different cycling kinetics." I think it is worth explicitly restating that this conclusion is drawn by analysing a data-set from which the "never-dividers" were removed. I appreciate the authors response that the "never-dividers" did not show a bias towards death or division. However, the concern in my first review is that, excluding cells that do not divide during the experiment may remove a small subpopulation (~5% of the total population) for which the IMT and AT are not well-described by a single distribution. I appreciate that the behaviour of these cells is both right and left censored and so appropriately accounting for them in the likelihood can be difficult. However, as the main result in this section is that the entire population of cells follows a similar distribution, I think that excluding the 5% of "never-dividers" is not well-justified and should be explicitly stated as a caveat on the quoted

conclusion.

Alternatively, I would be very happy to see the authors include these "never-dividers" in their analysis.

Response: We have now explicitly mentioned the exclusion of the never-dividers in two places in the first Results section.

In Model M3, on lines 191-192, once the fate decision has been made, is the accumulated life-span of the individual cell considered when sampling the "waiting time" from the appropriate IMT/AT distribution? It seems to me that this is necessarily a conditional probability, as the cell has survived some non-zero time without death/division, which should be accounted for when determining the fate waiting time. Once again, here, I think the manuscript could be improved by including more technical details, particularly given PLOS Comp Biol's readership.

Response: We thank the Reviewer for raising this important point. We have now clarified this by adding more details in the Main Text methods section. The accumulated life span is indeed considered because the hazards calculated for each cell are age-dependent. In both Models *M0* and *M3*, the hazard before drug is simply the division hazard, which changes as the cell ages from the time of birth. After drug, in Model M0 the total hazard is given by division + death hazards, which are again age dependent, where the cell ages continuously across the drug dosing time point. For Model *M3*, either the division or death hazard is used post drug, depending on which fate is decided for a particular cell.

Reviewer #3: I thank the authors for their careful and thorough responses to my previous comments.

Overall, I am satisfied with the revisions made to the manuscript, which adequately address the concerns I raised.

I have no further comments to raise and therefore support the publication of this work in PLOS Computational Biology.

Response: We thank the Reviewer for the positive comments on our revised manuscript.
